# Mesoporous Networks of *N*-Vinylpyrrolidone with (di)Methacrylates as Precursors of Ecological Molecular Imprinted Polymers

**DOI:** 10.3390/ma14226757

**Published:** 2021-11-09

**Authors:** Svetlana V. Kurmaz, Natalia V. Fadeeva, Anna I. Gorshkova, Sergey A. Kurochkin, Eugenia I. Knerelman, Galina I. Davydova, Vladimir I. Torbov, Nadezhda N. Dremova, Dmitry V. Konev, Vladimir A. Kurmaz, Vladislav M. Ignatiev, Nina S. Emelyanova

**Affiliations:** 1 Institute of Problems of Chemical Physics, Russian Academy of Sciences, Prosp. Akad. Semenova 1, 142432 Chernogolovka, Russia; natali-vi@inbox.ru (N.V.F.); anhen.gor@mail.ru (A.I.G.); oligo@icp.ac.ru (S.A.K.); kge@icp.ac.ru (E.I.K.); roxen67@mail.ru (G.I.D.); torbov@icp.ac.ru (V.I.T.); dremova@icp.ac.ru (N.N.D.); dkfrvzh@gmail.com (D.V.K.); kurmaz@icp.ac.ru (V.A.K.); ignvm@74.ru (V.M.I.); n_emel@mail.ru (N.S.E.); 2 Department of Fundamental Physical and Chemical Engineering, M.V. Lomonosov Moscow State University, Leninskie Gory 1, 119991 Moscow, Russia; 3 Faculty of Fundamental Sciences, Bauman Moscow State Technical University, Baumanskaya 2nd 5, 105005 Moscow, Russia

**Keywords:** *N*-vinylpyrrolidone, (di)methacrylates, porogens, nanopores, Rose Bengal, sorption, cyclic voltammetry, quantum chemical modeling, hydrogen bond

## Abstract

Mesoporous polymer networks were prepared via the cross-linking radical copolymerization of non-toxic hydrophilic *N*-vinylpyrrolidone (VP) with triethylene glycol dimethacrylate (TEGDM) and poly(ethylene glycol) methyl ester methacrylate (PEGMMA) in bulk, using appropriate soluble and thermodynamically compatible macromolecular additives with a branched structure as porogens. The branched copolymers of various monomer compositions were obtained by radical copolymerization in toluene, controlled by 1-decanethiol, and these materials were characterized by a wide set of physical chemical methods. The specific surface areas and surface morphology of the polymer networks were determined by nitrogen low-temperature adsorption or Rose Bengal (RB) sorption, depending on the copolymer compositions and scanning electron microscopy. The electrochemical properties of RB before and after its encapsulation into a branched VP copolymer were studied on a glassy carbon electrode and the interaction between these substances was observed. Quantum chemical modeling of RB-VP or RB-copolymer complexes has been carried out and sufficiently strong hydrogen bonds were found in these systems. The experimental and modeling data demonstrate the high potency of such mesoporous polymer networks as precursors of molecularly imprinted polymers for the recognition of fluorescent dyes as nanomarkers for biomedical practice.

## 1. Introduction

One of the general trends in polymer chemistry is the creation of molecularly imprinted polymers capable of recognizing template molecules or molecules of a similar structure which can occur as a result of shape, size and due to the interaction between the functional groups of the template and the polymer. G. Wulff and A. Sarhan reported on molecularly imprinted systems, and the process of their synthesis has been called molecular imprinting [1]. Taking into account the possible interactions between template and monomers, three approaches have been developed known as covalent, semi-covalent and a non-covalent imprinting. The idea of forming non-covalent prepolymerization complexes between a template and functional monomers was proposed by M. Mosbach and R. Arshadi [2]. The bond between the template and components of the mixture occurs due to the formation of hydrogen bonds, Van der Waals forces, etc. The template molecule is removed from a polymer matrix through repeated extraction with a solvent or a mixture of various solvents. In the case of non-covalent imprinting, the remainder of the template in the MIP is very small, and re-binding occurs due to the same interactions. It is the most effective and widely used approach to the preparation of MIPs due to its experimental simplicity, the easy removal of the template under mild conditions, fast template binding and release kinetics.

As a result of MIPs developments, synthetic polymeric receptors for various organic molecules including pesticides, medical compounds, amino acids, steroids, oligopeptides, etc. can be obtained and “smart” polymers can now be used in various application areas of biology and chemistry, namely, in selective separation and sample preparation [3], nanocomposite materials [4], biological assays [5,6], catalysis [7], drug delivery [8,9], sensors [10] etc. MIP_S_ are promising selective sorbents that can be used to clean up pollutants in water. A perspective approach to MIPs used in aqueous media can be associated with hydrophilic functional monomers and cross-linkers [11,12,13,14,15,16]. As a result, outstanding performance of imprinted polymers can be achieved by nonspecific binding diminution without complex surface modification [17].

It should be noted that porogenic solvents play an important role in MIPs fabrication. They are responsible for the creation of cavities in the polymeric matrix, and their pore size depends on the nature and solubility of porogen. Meso- and macro-pores in a polymer matrix are preformed, compared with micropores that slow down the diffusion process. Various strategies for the synthesis of a wide range of porous structures are available [18] including the molecular design of the initial monomers, self-assembly and/or the use of templates (preformed pores) leading to the formation of micro-, meso-, and macro-porous structures. For the creation of polymer materials with nanosized pores, dendrimers have been used as alternative templates and pore-directing agents for the synthesis of micro- and meso-porous structures [19]. However, their synthesis and isolation is a complex task, and their functionalization with appropriate groups is necessary for ensuring compatibility with various types of monomers.

The branched copolymers which are based on vinyl(idene) monomers as macromolecular porogens are highly attractive for use in the preparation of porous structures [20,21]. The sizes and density of branching in their macromolecules can be altered by changing the monomer, branching agent ratio and the conditions of radical copolymerization. These inert polymers are extracted from the polymer composite with a solvent, which does not significantly change their physical and chemical parameters and they can therefore be used as porogens repeatedly. Consequently, we assume that we are able to use the soluble copolymers of N-vinylpyrrolidone (VP) as macromolecular porogens [22,23,24] for the fabrication of polymer networks based on non-toxic hydrophilic VP as potential MIPs, designed for the recognition of pollutants and their removal from water. These branched copolymers consist of the same monomer units as the initial comonomer mixture, which forms the polymer network. This makes it possible to ensure their solubility in monomeric mixtures of VP and triethylene glycol dimethacrylate (TEGDM) of various compositions and to control the thermodynamic compatibility by varying their structure/properties without an additional modification, or the use of the solvents leading to the formation of macropores. The advantage of branched macromolecules over linear analogs of the same molecular weight include low viscosity in solutions, which makes it possible to obtain the monomer-polymer mixtures with low viscosity even at a significant content (20 wt% or more) of the polymer additive.

The final structure and properties of the polymer matrices, their specific surface area, swelling and mechanical stability will be defined by the degree of cross-linking, thermodynamic affinity and the size of the macromolecular porogen, as well as by the processes of phase separation induced by three-dimensional radical copolymerization (TRCP). The polymer matrices, as potential MIPs to remove the pollutants in water, in turn, should have an affinity for imprint molecules and be able to adsorb/desorb them from aqueous solutions and to bond with their functional groups through non-covalent interactions.

The aims of the present work are as follows: to prepare stable nanoporous polymer networks based on non-toxic VP through the use of nanostructured branched copolymers as macromolecular porogens (preformed pores); to study their adsorption and desorption properties related to Rose Bengal (RB) as a hydrophilic dye of the fluorescein family (biological object nanomarker); to understand the interaction between VP copolymer by methods of electrochemistry and to fulfill a quantum chemical modeling of the structure of the dye-monomer and dye-copolymer complexes. The issue of the macromolecular design of two types of the polymer matrices—VP-TEGDM and VP-PEGMMA-TEGDM nanoporous network copolymers—is considered and resolved using macromolecular additives of the appropriate composition, thereby ensuring their solubility and compatibility with the monomer mixtures. This is expected to increase their polarity and affinity to the hydrophilic dye and water by introducing PEGMMA units with *M*_n_ of 500 g/mol into the polymer chains.

## 2. Materials and Methods

### 2.1. Reagents

The monomer, *N*-vinylpyrrolidone (VP, Alfa Aesar, Ward Hill, MA, USA), was purified by vacuum distillation to remove the NaOH inhibitor. Triethylene glycol dimethacrylate (TEGDM, Aldrich, St. Louis, MO, USA), poly(ethylene glycol) methyl ester methacrylate (PEGMMA, Aldrich), 1-decanethiol as a chain transfer agent (DT, Alfa Aesar), isopropyl alcohol (IPA, Khimmed, Moscow, Russia) of the extra purity grade as well as Na_2_HPO_4_ and NaH_2_PO_4_ were used without additional purification. The initiator, azobisisobutyronitrile (AIBN), was purified through its recrystallization from ethanol.

To study the sorption properties of polymer matrices, a hydrophilic dye, Rose Bengal sodium or Disodium 3,4,5,6-tetrachloro-2- (2,4,5,7-tetraiodo-6-oxido-3-oxoxanthen-9-yl) benzoate, Acid Red 94, (RB) (Alfa Aesar), was used without preliminary purification.

### 2.2. Preparation of Macromolecular Porogens—VP-TEGDM and VP-PEGMMA-TEGDM Branched Copolymers

Macromolecular porogens—br-VP-TEGDM and br-VP-PEGMMA-TEGDM copolymers—were obtained by radical copolymerization in toluene (75 vol.%) under chain transfer to 1-decanethiol at the molar ratios of VP:TEGDM: DT and VP: PEGMMA: TEGDM: DT equal to 100:12:12 and 95:5:5:5, as described in [25]. The concentration of AIBN was 0.01 mol/L. The copolymers were isolated from the solution by precipitation with a tenfold excess of *n*-hexane and dried to a constant weight in vacuum. The yield of the high molecular weight fractions of the copolymers were ~90%. These were the only fractions used in the study.

### 2.3. Preparation of VP-TEGDM Polymer Composites with br-VP-TEGDM Polymer Additive

The br-VP-TEGDM copolymer (20 wt%) was dissolved in the VP-TEGDM monomer mixture (40:60 wt%) for a duration of 3 days, obtaining a visually transparent monomer-polymer mixture, then three-dimensional radical copolymerization at 60 °C, initiated by AIBN, was carried out in glass ampoules that were 2.5 mm in diameter. After the copolymerization of the monomer-polymer mixture, opalescent block samples were obtained.

### 2.4. Preparation of VP-PEGMMA-TEGDM Polymer Composites with br-VP-PEGMMA-TEGDM Additive

The br-VP-PEGMMA-TEGDM copolymer (20 wt%) was dissolved in the monomer mixtures of VP-PEGMMA-TEGDM for various compositions (40:5:55, 40:10:50, and 40:20:40 wt%) over several days, through which visually transparent monomer-polymer mixtures were obtained, then a three-dimensional radical copolymerization at 60 °C, initiated by AIBN, was carried out in glass ampoules that were 4 mm in diameter. After the copolymerization of the monomer-polymer mixtures, block samples were obtained. The polymer composites obtained from the monomer mixtures with 10 and 20 wt% PEGMMA were uniformly opalescent, in contrast to the sample with 5% PEGMMA, which had regions with different transparency. Due to the high degree of inhomogeneity, this sample was not investigated.

### 2.5. Extraction of Sol Fractions from the Polymer Composites

The extraction of macromolecular additives from the obtained polymer composites was carried out using isopropyl alcohol at its boiling point (80 °C) in a Soxhlet apparatus, or at room temperature (25 °C). The obtained sols (soluble part) and gels (insoluble part) were dried in vacuo to achieve a constant weight.

### 2.6. IR- and ^1^H NMR Spectroscopy

The copolymers that were used as macromolecular additives were identified using IR- and ^1^H NMR-spectroscopy. The IR spectra of the copolymers were recorded on a Bruker α FTIR instrument in the transmission mode. The ^1^H NMR spectra of the copolymers in deuterated chloroform (6 mg/mL) were recorded on an AVANCE III 500 MHz BRUKER BioSpin superconducting pulsed broadband two-channel NMR spectrometer using glass ampoules that were 5 mm in diameter.

### 2.7. Elemental Analysis

The monomer composition of the branched copolymers was determined using elemental analysis on a CHNS/O Vario Micro cube analyzer (Elementar GmbH, Langenselbold, Germany).

### 2.8. Size-Exclusion Chromatography

The molecular weight of br-VP-TEGDM was measured by size-exclusion chromatography (SEC) using a Waters GPCV 2000 liquid chromatograph (2 PS-gel columns, 5 μm, MIXED-C, 300 × 7.5 mm^2^), equipped with a refractometric detector (RI) and a WYATT DAWN HELEOS II light scattering detector, *λ* = 658 nm (MALLS). We used the Software Empower Pro and Astra (5.3.2.20 version). *N*-methylpyrrolidone with 1 wt% of LiCl was used as eluent and its measurement temperature was 70 °C.

### 2.9. Transmission Electron Microscopy

The TEM-image of br-VP-TEGDM was obtained from the aqueous solution (0.1 mg/mL) using Leo 912 AB equipment. Phosphotungstic acid was used to contrast the sample.

### 2.10. Dynamic Light Scattering

The effective absolute molecular weight of scattering centers and the second virial coefficient *A*_2_ of the br-VP-PEGMMA-TEGDM copolymer in water were determined using the Debye method at 22 °C, from the light scattering data of solutions obtained on a Photocor Compact (LTD Photocor, Moscow, Russia), equipped with a diode laser operating at 654 nm. The increment of the refractive index d*n*/d*c* was calculated for the terpolymer solution in water (1–10 mg/mL). The hydrodynamic radius *R*_h_ of the terpolymer in isopropyl alcohol and water was determined at a detection angle of 90°. The solutions were filtered in advance using a filter with pore diameter of 0.45 μm. Before the measurements were obtained, the vials with the solution were thermostated for ~20 min. The scattered light autocorrelation functions determined in the dynamic light scattering mode were processed using “DynaLS” software. The value of the hydrodynamic radius *R*_h_ of scattering centers was calculated using the Stokes-Einstein equation *D* = *kT*/6πη*R*, where *D* is the diffusion coefficient, *k* is the Boltzmann constant, *T* is the absolute temperature, and η is the viscosity of the medium, in which dispersed particles were suspended.

### 2.11. Differential Scanning Calorimetry

The glass transition temperature of the branched copolymer, VP-TEGDM copolymer composite and corresponding cross-linked copolymer was determined using differential scanning calorimetry on a METTLER TOLEDO STAR^e^ DSC 822 module. For this purpose, the samples were placed in closed aluminum cells and measured in the temperature range from 0 to 180 °C at a heating rate of 5 °C/min. The desired value was considered to be the temperature determined in the third measurement cycle in the heating—cooling mode, in accordance with the DIN standard.

### 2.12. Physical Mechanical Measurements

The physical mechanical properties of the VP-TEGDM cross-linked copolymer, the corresponding polymer composite, and the porous polymer matrix were studied on a Zwick/Roel universal material testing machine. Cylindrical samples with a size of ~5 × 3 mm^2^ were used.

### 2.13. Scanning Electron Microscopy

The surfaces of the VP-TEGDM and VP-PEGMMA-TEGDM porous polymer matrices, after the extraction of the copolymer additives, were examined using scanning electron microscopy on a Zeiss Leo Supra 25 instrument. For this purpose, ultrafine carbon was spayed on the end surface of the cylindrical samples.

### 2.14. Measurement of the Specific Surface Area of Porous Polymer Matrices

*The BET analysis.* The samples of the porous cross-linked copolymers were preliminarily evacuated at ca. 80 °C and investigated by low temperature nitrogen adsorption on an ‘Autosorb-1′ analyzer (Quantachome Instruments Corp., Boynton Beach, FL, USA). The specific surface area (*S*_sp_) of these copolymers were determined using the Brunauer-Emmett-Teller (BET) equation

1W×[(P0P)−1]=1Wmax×C+C−1Wmax×C×(PP0)

where *P* is the nitrogen pressure in the sample cell, *P*_0_ is the saturated nitrogen vapor pressure at 77 K, *W* is the weight of nitrogen absorbed at a given value of *P*/*P*_0_, *W*_m_ is the weight of the adsorbate in the surface monolayer, *C* is the parameter of the BET equation characterizing the adsorbent—adsorbate interaction. The value of the total pore volume, *V*_p_ was determined by measuring the amount of nitrogen adsorbed at the value of *P*/*P*_0_ close to 1. The pore size distribution curves were plotted using the Barrett, Joyner, and Halenda (BJH) method. The error in the determination of *V*_p_ and *S*_sp_ values did not exceed 7%.

### 2.15. Electronic Absorption Spectroscopy

The absorption spectra of aqueous solutions of RB during adsorption and desorption were recorded on a SPEKS SSP-705-1 spectrophotometer (Russia) using cuvettes of 0.2 cm or 1 cm thickness.

### 2.16. Electrochemical Studies

Electrochemical experiments were carried out in a three-electrode 10 cm^3^ glass cell without the separation of the cathodic and anodic areas via cyclic voltammetry and chronoamperometry using an Autolab/PGSTAT302N universal high-speed potentiostat/galvanostat (ECOCHEMIE, Utrecht, The Netherlands). The range of the potential scan rate was 0.05–0.5 V s^−1^. The working electrodes were glassy carbon (GC), Pt and Au discs with a diameter of 1 mm, soldered into a glass. The auxiliary electrode was a Pt wire, and the reference electrode was a saturated silver/silver chloride electrode (Ag/AgCl) in aqueous solutions.

### 2.17. Quantum Chemical Modeling of the Structure of RB Complexes with the VP Monomer and a VP-VP-VP Site of the Copolymer

Quantum chemical calculations were performed within the framework of the density functional theory (DFT), with the full optimization of the geometry of the initial molecules and their complexes in the Gaussian 09 program [26]. The TPSSh hybrid functional [27] and the 6–31G *//6–311++G** basis set were used as the method and as a basis. The influence of the solvent (water) was also taken into account using the polarizable continuum model (PCM). Earlier, this combination of calculating methods allowed us to obtain [28] a good correlation between the results of the modeling of such objects and the experimental data. There are no imaginary vibrational frequencies in the calculation results, and all the optimized structures correspond to the minimum potential energy. Parameters such as the energy of the secondary interactions, the Wiberg index and the bond order were obtained from calculations using natural basic orbitals (NBO).

For the analysis of wave functions using the QTAIM method, the AIMALL software package (version 10.05.04) [29] was used. The wave functions of the structures were calculated in the same approximations as the optimization of geometry. In particular, from the analysis of the wave functions, the energies of intermolecular bonds (*E*_b_), the electron density (ρ), and the Laplacian of the electron density (∇^2^ρ) were found at the critical points of the bond. The energies of intermolecular bonds were calculated using the formula *E*_a-b_ ≈ 1/2*ν*_e_(*r*) [30], where *E*_a-b_ is the A-B bond energy, and ν_e_(*r*) is the potential energy density at the critical point of the A-B bond. The illustrations were made using ChemCraft program (version 1.8) [31].

### 2.18. Preparation of VP-*TEGDM* Porous Copolymers for Studying RB Adsorption from Aqueous Solutions

Opaque (milky white) samples 1 and 2 of the VP-TEGDM cross-linked copolymers were placed in 5 mL of water for 3 days at room temperature. Then they were air dried. The masses of the samples for the RB adsorption studying were 0.020 and 0.019 g, respectively.

### 2.19. Preparation of VP-PEGMMA-*TEGDM* Porous Copolymers for Studying RB Adsorption from Aqueous Solutions

In the experiments, the VP-PEGMMA-TEGDM porous polymer matrices of two compositions (40:10:50 and 40:20:40 wt%) were used. Before beginning the experiments on the RB adsorption, samples 3–5 (0.138, 0.080, and 0.082 g in weight, respectively) were placed in water for 3 days at room temperature. In this case, samples 3 and 4 swelled slightly and remained intact, while sample 5 collapsed. After drying in air, the weights of the samples 3 and 4 were 0.1214 and 0.0704 g, i.e., the weight loss in the both cases was about 12%, possibly as a result of further extraction of water-soluble fractions from the samples.

### 2.20. Experimental Procedure for RB Adsorption from Aqueous Solutions by Porous Polymer Matrices

A solution of RB was prepared in deionized water (0.55 × 10^−4^ M) and its absorption spectrum was recorded in a 0.2 cm thick cuvette. The value of the optical density of the RB absorption band at 544 nm was taken as an initial value *A*_0_. Samples 1 and 2 were placed into glass vials, and 3 mL of the aqueous RB solution was added. The absorption spectra of the aqueous RB solutions were recorded at certain time intervals. The maximum value of the RB absorption band in each measurement also corresponded to *A*_t_. The adsorption degree of RB by the polymer matrix was calculated using the following formula: *S*_ads_ = (*A*_0_ − *A_t_*)/*A*_0_ × 100%, and the dependence of the *S*_ads_ value on time, *t* was plotted in the coordinates of the Fick equation.

The experimental procedure for RB adsorption from aqueous solutions was performed using VP-PEGMMA-TEGDM porous polymer matrices of various compositions. Samples 3 (0.0851 g), 4 (0.0708 g), and 5 (0.0522 g) were placed into glass vials, and 3 mL of the RB solution (0.55 × 10^−4^ M) was added. The optical density of the RB absorption band was recorded in a 0.2 cm thick cuvette. The value at the maximum of the absorption band in each measurement corresponded to *A_t_*. The value of adsorption degree, *S*_ads_ was calculated, and the dependence of the *S*_ads_ value on time, *t* was plotted for the coordinates of the Fick equation.

### 2.21. Experimental Technique for RB Desorption from VP-*TEGDM* Polymer Matrices in Aqueous Solutions

To study the RB desorption from the VP-TEGDM porous polymer matrices, 5 mL of water was poured into vials that contained samples 1 and 2, and the process of the RB desorption was recorded by changing the absorption spectra of the aqueous solutions over time, using a 1 cm thick cuvette. The value of *S*_des_ at the corresponding time was calculated according to the formula:

*S*_des_ = *A*_des_/Δ*A*_ads_ × 100%

where Δ*A*_ads_ is the difference in the optical densities of the aqueous dye solution before and after adsorption and *A*_des_ is the optical density of the aqueous dye solution. The dependence of the *S*_des_ value on time was plotted using the coordinates of the Fick equation.

### 2.22. Experimental Technique for RB Desorption from VP-PEGMMA-*TEGDM* Polymer Matrices in Aqueous Solutions

To study RB desorption, 5 mL of water was poured into vials with samples and the process of desorption of the dye was recorded by altering the absorption spectra of the aqueous solutions over time using a 1 cm thick cuvette. The value of *S*_des_ was calculated, and the dependence of *S*_des_ on time was plotted in the coordinates of the Fick equation.

### 2.23. Determination of the Specific Surface Area from the Data of RB Adsorption by Polymer Matrices

The specific surface areas of the samples were calculated as described in [32]. In order to do so, the amount of the dye ν (mol) adsorbed by the sample was determined using the difference in the optical densities of the initial aqueous RB solution and the RB solution after the completion of adsorption (respectively, Δ*A*_ads_, *A*_0_, and *A*_ads_):

ν = Δ*CV* = (*A*_0_ − *A*_ads_) *V*/(ε*l*) = Δ*A*_ads_*V*/(ε*l*)

where Δ*C* is the change in concentration in the solution; mol L^−1^; *V* is the volume of the solution, L; *l* is the thickness of the cuvette (optical path length), cm and *ε*~8.7 × 10^4^ L mol^−1^ cm^−1^ is the molar absorption coefficient of the aqueous RB solution at a maximum wavelength of 544 nm calculated from the dependence *A*(*C*) for the RB solutions of various concentrations C.

The value of *S*_sp_ of the polymer network was calculated using the following formula:

*S*_sp_ = (π*r*_ef_^2^*N*_A_*ν*)/*m*

where *r*_ef_ = 1.28 × 10^−9^ m is the effective radius of the RB molecule [33]; *N*_A_ is the Avogadro’s number; *ν* is the amount of absorbed dye, mol and *m* is the mass of the sample (g).

## 3. Results and Discussion

### 3.1. Synthesis and Characteristics of Branched Copolymers of VP as Potential Macromolecular Porogens during the Formation of Polymer Matrices

**Figure 1:**
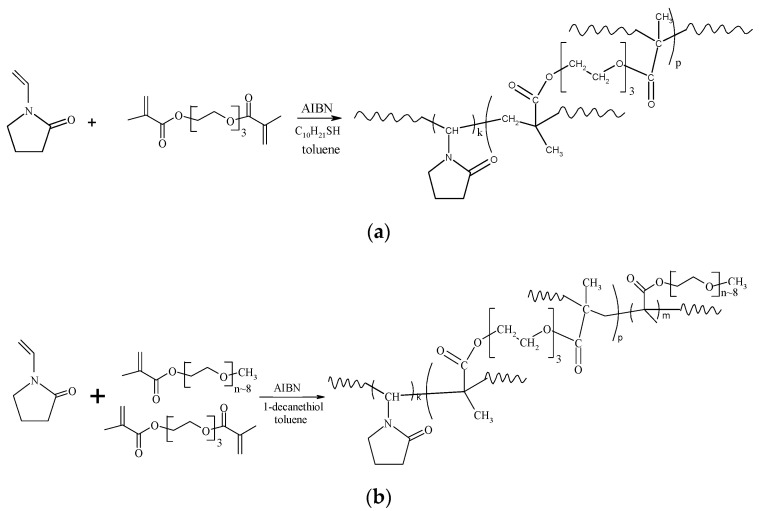
Scheme of the synthesis of the br-VP-TEGDM (**a**) and br-VP-PEGMMA-TEGDM (**b**) copolymers.

The br-VP-TEGDM and br-VP-PEGMMA-TEGDM copolymers were obtained via their radical copolymerization in toluene, controlled by a chain transfer agent, 1-decanethiol, according to the scheme provided (Figure 1).

During radical polymerization, one of the double bonds of TEGDM, as a branching agent, participates in the growth of the main chain, and the other one—in formation of side branches, appears when polymer radicals are attached to its “pendant” C = C bond. Within the primary polymer chain, the “pendant” C = C bonds can interact inside the molecular chain to form cyclic structures [34]. However, during the copolymerization of VP with PEGMMA and TEGDM, the comonomer with a bulky substituent appears to create steric hindrances for intramolecular cyclization and the formation of nano- and microgel particles. PEGMMA units, in turn, also limit the occurrence of intermolecular crosslinking, leading to macrogel formation. The copolymerization of these monomers, under the chain transfer conditions at an equimolar ratio of the branching agent to DT, resulted in the formation of shorter primary polymer chains.

**Figure 2:**
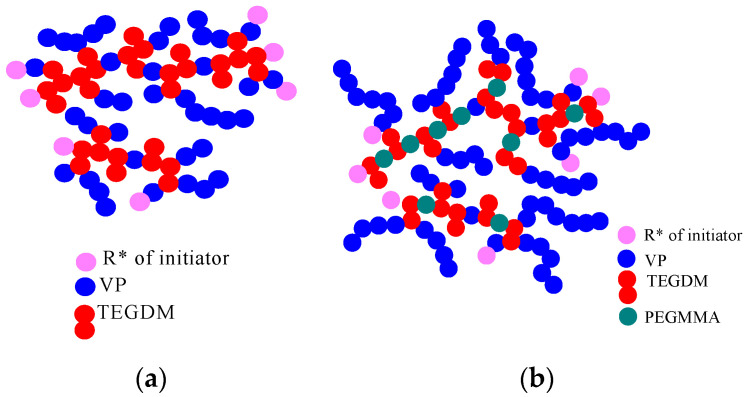
Schematic representation of possible topological structures of the copolymer br-VP-TEGDM (**a**) and br-VP-PEGMMA-TEGDM (**b**) copolymers.

TEGDM appeared to be more active in radical copolymerization as compared to VP [25]. This indicates that PEGMMA and TEGDM have a similar type of reactivity. As a result, growing radicals of all types experience the addition of predominantly methacrylic monomers. After the copolymerization of active methacrylic monomers, a structure with side double bonds of TEGDM is formed and PVP chains are primarily attached to the “pendant” C = C bonds. The possible topological structures of the br-VP-TEGDM and br-VP-PEGMMA-TEGDM copolymers are shown in Figure 2. Under the conditions of the chain transfer to DT, and as a consequence, the limiting of the intermolecular crosslinking reaction and the formation of a macrogel, polymer products were obtained with a yield of more than 80%. They were soluble in both polar and low-polarity media including water, alcohols, N-methylpyrrolidone, and VP-(di)methacrylate monomer mixtures.

### 3.2. FTIR and ^1^H NMR Spectroscopy Data Indicate the Monomeric Composition of the Copolymers

**Figure 3:**
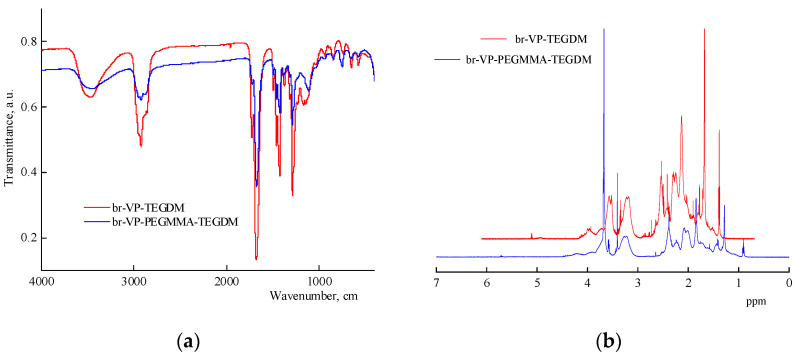
IR spectra (**a**) and ^1^H NMR (**b**) of br-VP-TEGDM and br-VP-PEGMMA-TEGDM copolymers, respectively.

According to the FTIR spectroscopy data (Figure 3a), the copolymers consist of VP and (di)methacrylate units. A characteristic feature of the FTIR spectrum of the ternary copolymer is that the absorption band at 1100 cm^−1^, corresponding to the vibrations of -C-O- ether bonds in the PEGMMA units of the terpolymer, and the absorption bands at 1720 and 1650 cm^−1^ are found to be related to C=O groups in the methacrylates and the VP units, respectively. The intensity of the absorption bands in the region of 3000–2800 cm^−1^, related to the stretching vibrations of the –CH_2_- groups, increases due to the appearance of the PEGMMA units and DT residues in terpolymer chains. A broad peak with a maximum at 3500 cm^−1^ corresponds to the stretching vibrations of the OH group of water sorbed by the amphiphilic copolymers. According to the ^1^H NMR data (Figure 3b), the copolymers contain residual double bonds of TEGDM, and protons in the –SC_10_H_21_ group of the DT residue in the polymer chains are therefore identified. The ^1^H NMR spectra of the terpolymers contain PEGMMA proton signals in the range of 3.4–3.9 ppm (Figure 3b). In addition, two groups of the bands related to VP units were observed for the terpolymers at δ 3.0–4.0 ppm and δ 1.4–2.4 ppm. Signals, resulting from protons of a CH_3_- group in the TEGDM units, were detected in the spectrum of the terpolymers at 0.9 ppm. Signals at 1.3 and 2.5 ppm may correspond to the protons of -(CH_2_)_8_- and CH_2_-S-fragments of DT. In the NMR spectrum, a broad band of 4.1 ppm is observed, which corresponds with the hydrogen atoms in the –CH_2_–CH_2_– fragment of the reacted TEGDM. However, weak signals of protons are observed at the double bond of TEGDM, within the range of 6.5–5.0 ppm. They should be attributed to the unreacted C = C bonds of TEGDM units.

The molar composition of the obtained copolymers was calculated using the elemental analysis data (Table 1). It can be seen that the copolymers have a similar monomer composition but differ in the content of DT residues in the polymer chains.

Table 1 shows the absolute weight-average molecular weight, *M*_w_, and the polydispersity index *p* of br-VP-TEGDM, indicating a rather narrow molecular weight distribution. The Zimm factor *g*’, calculated as a ratio of the root-mean-square radius of the gyration of macromolecules with *M*_w_~10^4^ to that of linear PVP, was measured as 0.6–0.7 and indicated their branching character. The branched copolymer is a set of macromolecules with different topological structures. It is a result of the analysis of the dependences of the molecular weight *M*_w_ on the eluent volume *V*_R_ for the VP-TEGDM copolymer and linear PVP. Due to the high content of end chains, the br-VP-TEGDM copolymer had a low glass transition temperature, *T*_g_.

The physicochemical characteristics of br-VP-PEGMMA-TEGDM are also shown in Table 1. The values of its absolute molecular weight, *M*_w_ and *T*_g_, are similar to those of br-VP-TEGDM. Despite the inclusion of DT residues in polymer chains, this copolymer presents a high thermodynamic affinity for water, as observed from the value of *A*_2_, equal to 9.0 × 10^−4^ cm^3^ × mol/g^2^.

**Figure 4:**
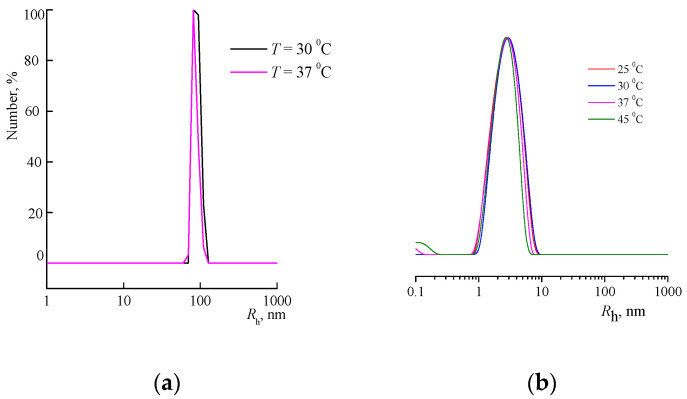
DLS curves of the br-VP-TEGDM copolymer in the aqueous solution (**a**) at various temperatures, and the dependences of the light scattering intensity on the size of scattering centers of br-VP-PEGMMA-TEGDM in IPA (**b**).

Both copolymers are composed of monomers with different polarities. VP is a typical polar monomer, whereas TEGDM and PEGMMA are low-polar monomers, and the –SC_10_H_21_ groups are non-polar fragments of the chain transfer agent. The hydrophilic-hydrophobic balance of the macromolecules is determined by their ratio and, in turn, regulates their behavior in polar media such as water and isopropyl alcohol. As a result of the self-assembly of these solvents, they are able form stable aggregates. So, the br-VP-TEGDM copolymer exists in water as a unimodal aggregate with an *R*_h_ of about 100 nm at the peak maximum (Figure 4a). The scattering centers of the individual br-VP-PEGMMA-TEGDM macromolecules, with an *R*_h_ of ca. 3 nm, were present in the alcohol solution at a concentration of 20 mg/mL (Figure 4b). However, in water, at a concentration of 10 mg/mL, they are around 4 nm in size, and their contribution to light scattering is decisive. As can be seen from Figure 4b, in the range from 25 to 45 °C, the size of the scattering centers does not depend on temperature, i.e., the copolymer is heat-insensitive.

**Figure 5:**
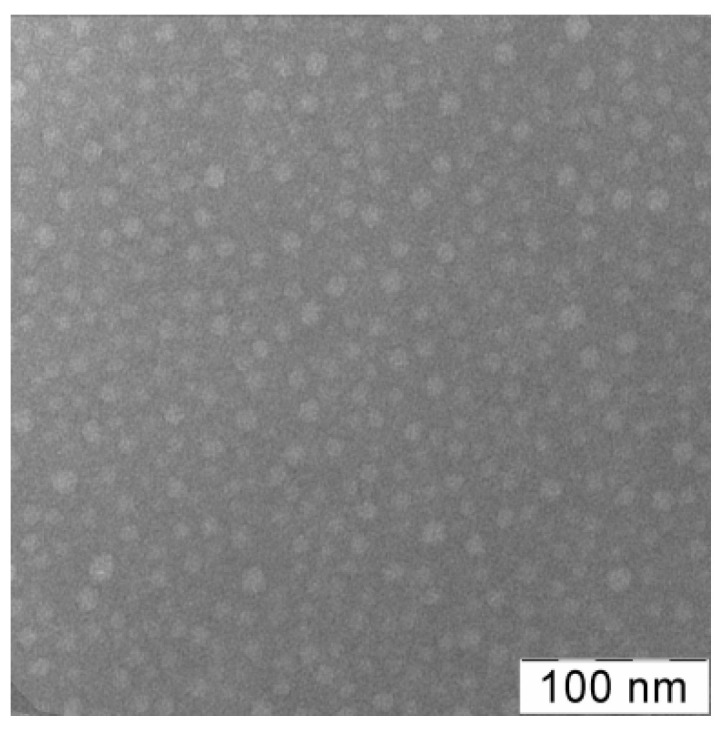
TEM-image of br-VP-TEGDM copolymer.

The micrograph (Figure 5) reveals the presence of spherical particles of the br-VP-TEGDM copolymer with a diameter of 10–12 nm. It becomes apparent that, as a result of collapsing the polar shell, the particles are of a smaller size than in aqueous solutions.

Furthermore, it is important to understand the dynamic characteristics of the polymer additives as potential porogens (preformed pores) in the corresponding monomer mixtures in order to predict pore sizes in the polymer matrix. In this regard, using the br-VP-TEGDM copolymer as an example, we investigated light scattering of the monomer–polymer mixture, from which the polymer matrix was obtained. The intensity, I, of light scattering of the br-VP-TEGDM copolymer—VP monomer mixture at 20 °C was measured beforehand. The I value for the mixture was low in the range of copolymer concentrations of 1–10 wt%, which indicated a high solubility of the copolymer in the polar monomer and their thermodynamic compatibility. However, in the mixture of monomers of VP-TEGDM, with a composition of 40:60 wt%, even with the addition of low concentrations of the copolymer (up to 1%), there was a multiple increase of the I value from 9 × 10^3^ to (2–3) × 10^5^ cps. This may be due to the presence of aggregates in the mixture as a result of decreasing the polarity of the medium and the thermodynamic affinity of the components.

However, the br-VP-PEGMMA-TEGDM polymer additives did not significantly affect the *I* value of the corresponding monomer–polymer mixtures. The intensity of light scattering by the initial VP-PEGMMA-TEGDM monomer mixtures of various composition was in the range of (2–6) × 10^4^ cps, and for monomer mixtures with the 20% polymer additive it was in the range of (3–5) × 10^4^ cps, which indicated a high solubility of the polymer additive in these monomeric mixtures and their thermodynamic affinity.

In general, the VP-TEGDM and VP-PEGMMA-TEGDM monomer–polymer mixtures represented dispersed systems which were time-stable and did not separate into individual components. It can be assumed that during the formation of the polymer composites, the individual macromolecules and their aggregates will contain preformed pores and that after their removal, porous polymer matrixes will be obtained.

### 3.3. Formation of Polymer Composites and Obtaining Porous Polymer Matrices

Table 2 contains the compositions and designations of the monomer–polymer mixtures, the polymer composites and the final polymer matrices. The polymer matrix is a densely cross-linked chemical network, in which a dispersed phase, specifically the polymer additive, is distributed. During TRCP, a microphase separation occurs [34], and macromolecular additives (individual macromolecules and/or their aggregates) are released into separate regions. The changes in the optical properties of the polymer networks and the appearance of opalescence confirm the formation of microregions. The refractive index of the dispersed phase differed from that of the polymer matrix, and the polymer composites were opalescent. This is also confirmed by the fact that the VP-TEGDM polymer composite has two glass transition temperatures T_g_, determined by DSC at 58 and 125 °C, while the cross-linked copolymer has only one T_g_ equal to 124 °C. The T_g1_ value is close to the glass transition temperature of the br-VP-TEGDM copolymer (Table 1). The appearance of two glass transition temperatures in the copolymer composite is a result of the defrosting of the mobility of segments of the polymer additive, and, in turn, of the polymer matrix itself with increasing temperature.

The obtained polymer composites of various compositions were monoliths (block samples). By analyzing the slopes of the linear sections of deformation ε—stress σ curves, the elastic moduli E of the VP-TEGDM polymer composite and the VP-TEGDM cross-linked copolymer were found. The polymer composite was characterized by a lower value of E = 1.0 × 10^3^ MPa in comparison to the cross-linked copolymer with a higher value of E = 1.4 × 10^3^ MPa. A decrease in the elastic modulus of the polymer composite may be a result of the diminishing conversion of C = C bonds and the crosslinking density, as well as a plasticizing effect of the polymer additive, which weakens the intermolecular interactions of the polar groups of the macromolecules.

### 3.4. Sol-Gel Analysis of the Polymer Composites and Their Structures

Table S1 shows the results of the sol-gel analysis, carried out under different conditions, with VP-PEGMMA-TEGDM polymer composites of various compositions as an example. The total weight of the sol and the gel exceeded the weight of the initial samples, which may be due to the presence of isopropyl alcohol which are hydrogen-bonded to oxygen-containing groups of the copolymers.

**Figure 6:**
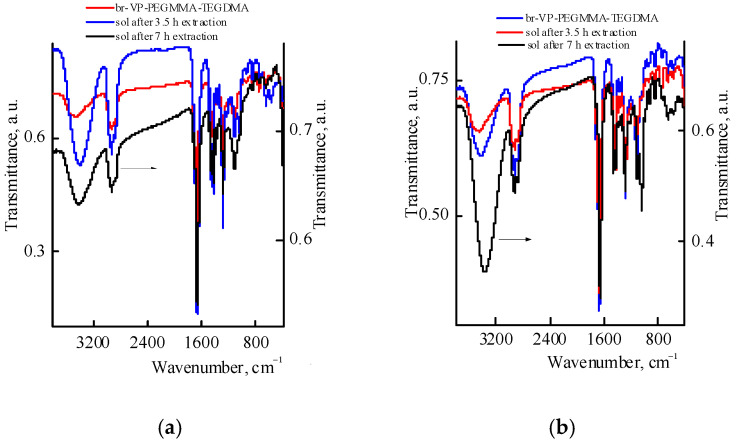
IR spectra of br-VP-PEGMMA-TEGDM copolymer and the sols isolated from the VP-PEGMMA-TEGDM polymer composites prepared from mixtures of (40:10:50): 20 (**a**) and (40:20:40): 20 wt.% composition (**b**).

An analysis of the IR spectra (Figure 6a) of the br-VP-PEGMMA-TEGDM copolymer and the sols isolated from the corresponding polymer composites confirms this assumption. In the IR spectra of the sols, the main absorption bands are characteristic of the copolymer, and are, in particular, related to the stretching vibrations of the C=O bond in the lactam cycle of VP units and the C=O bond of the (di)methacrylate units. However, new absorption bands appear in the spectrum at ~1040 and ~1110 cm^−1^, corresponding to the stretching vibrations of C–O bond in isopropanol. A shift in the absorption band of the C=O bond in VP units to lower values (Δν~18 cm^−1^) indicates the formation of the hydrogen bond between the solvent and the copolymer. From the comparison of the IR spectra of the sols isolated at different extraction times and the ratio of the optical densities of the absorption bands of the C=O groups of different monomer units, it can be concluded that the composition of the sol changes, and the yield of the macromolecules that are enriched with (di)methacrylates increases over time. Meanwhile, sols extracted from the VP-TEGDM polymer composites can be maintained in a high molecular weight product and a low-molecular oligomer VP (*M*_p_ ca. 500) that is formed after the depletion of active dimethacrylate in radical copolymerization [24]. As assumed is expected, the analyzed sols separated from the VP-PEGMMA-TEGDM polymer composites contain a branched copolymer, VP oligomer and the residual solvent.

Channels appear in the process of swelling of the polymer networks in a “good”, i.e., a thermodynamic suitable solvent, through which macromolecular additives enter the solution to form pores and channels of an open type. The swelling of the polymer network in alcohol and its drying can lead to the transformation of the pore system in the polymer matrix and, consequently, to the appearance of macropores and the collapse of small pores. However, a high density of crosslinking of the polymer network, a slight change in the size of the samples during swelling, and their integrity indicates that the porous structure is retained. This is evidenced by the following changes in their optical properties: the samples of the VP-TEGDM and VP-PEGMMA-TEGDM polymer matrices, after the extraction of the corresponding additives and drying, are opaque (milky-white) as a result of the appearance of a dispersed phase with a different refractive index. It should be noted that after the extraction of the polymer additive, the elastic modulus E of the VP-TEGDM polymer matrix was around 1.1 × 10^3^ MPa, i.e., the physical and mechanical properties of the chemical network remained practically unchanged.

Indirect evidence of the presence of pores in the polymer matrices is provided by the data on the kinetics of water vapor sorption by VP-TEGDM polymer composite and the corresponding porous polymer matrix (Figure S1) [22]. The process of water sorption by the polymer composite is accompanied by the release of water-soluble products and, as a consequence, the maximum value of the sorption of water vapor S does not exceed 10% and decreases with time (Figure S1). However, after removing the br-VP-TEGDM polymer additive, the sorption of water vapor sharply increases to ~40–50%, which is clearly a result of the presence of pores. The process of sorption occurs in accordance with the capillary mechanism and is reversible.

**Figure 7:**
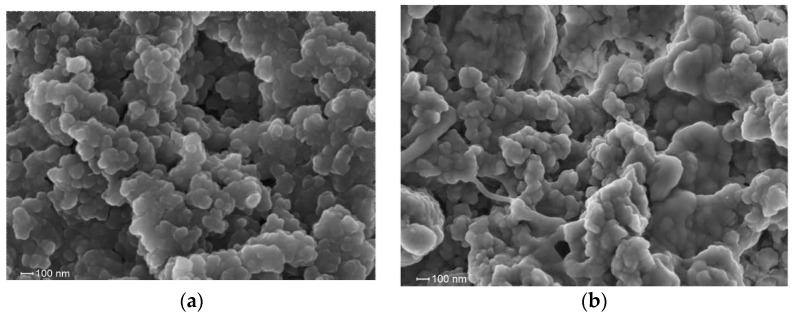
Micrographs of the VP-TEGDM polymer matrix after extraction of the polymer additive (**a**) and VP-TEGDM polymer matrix after RB adsorption study (**b**).

The results of the SEM study of the surface of the VP-TEGDM polymer matrix (end surface, cleavage) after the extraction of the copolymer additive confirm the presence of pores and a developed surface area (Figure 7a). The micrograph reveals open pores between the bound polymer particles that are of nanometer size and that the surface of the polymer matrix is granular.

**Figure 8:**
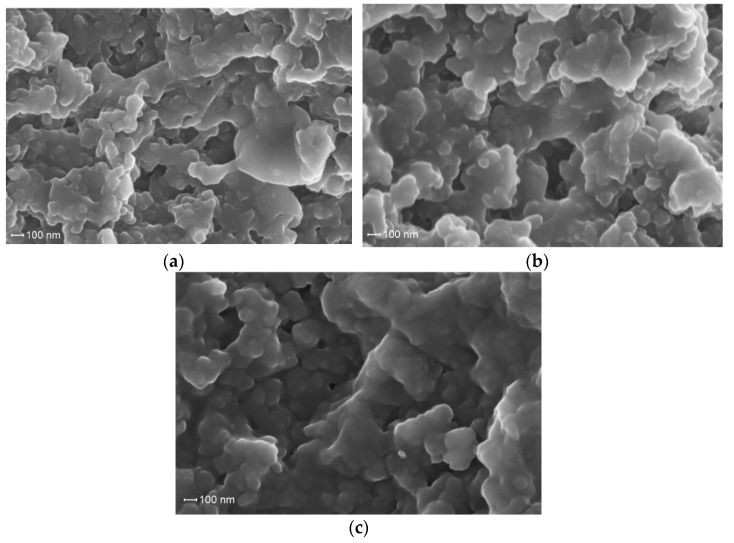
Micrographs of the VP-PEGMMA-TEGDM polymer matrices of various compositions after extraction of the polymer additive (samples 3–5, (**a**–**c**), respectively).

Figure 8 presents photomicrographs of the VP-PEGMMA-TEGDM polymer matrices of various compositions (samples 3–5), which also indicate their porous structure. However, the globular morphology of the polymer particles is less manifested in comparison to that of the VP-TEGDM polymer matrix, and, in this region, large agglomerates with fewer granularities are observed, which allows us to make an assumption about their less developed surface. The difference in the morphology of studied polymer matrixes may be a result of their various structural heterogeneity and supramolecular organization [25].

### 3.5. Analysis of the Porous Structure of Polymer Matrices by Low-Temperature Nitrogen Adsorption and Dye Adsorption from Aqueous Solution

The quantitative characteristics of the porosity of the obtained polymer matrices were determined by low-temperature nitrogen adsorption (Table 3) using the BET analysis. The S_sp_ value of the VP-TEGDM polymer matrix reached rather high values which are typical for polymer networks based on vinyl monomers prepared by the TRCP [35]. The VP-TEGDM cross-linked copolymer, obtained in the absence of a polymer additive, was non-porous and its S_sp_ value was less than 1 m^2^/g.

**Figure 9:**
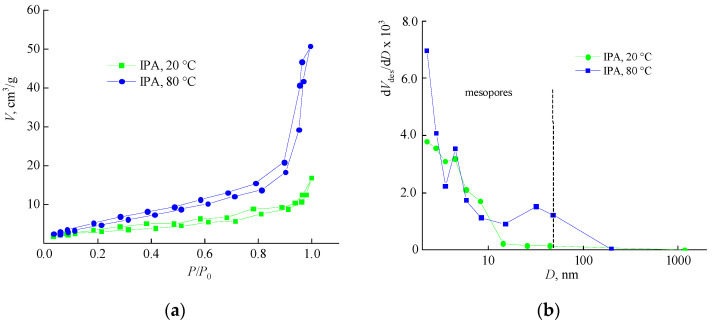
Adsorption-desorption isotherms (**a**) and pore size distribution curves (**b**) of the VP-TEGDM polymer matrix after IPA extraction of the polymer additive at 20 and 80 °C.

Figure 9 shows the adsorption-desorption isotherms and the pore size distribution curves of the VP-TEGDM polymeric matrices obtained at the boiling point of IPA and at room temperature. The isotherm shape belongs to the type IV characteristic for mesoporous bodies [36]. From the absence of a sharp rise in the isotherm at low values of *P*/*P*_0_, it can be concluded that there are no micropores. The presence of a hysteresis on the curves also indicates the presence of mesopores in the studied matrix. The isotherms of sample 1, extracted under mild conditions, are positioned below the corresponding curves for the sample subjected to a boiling solvent treatment. The distribution curves of both samples mainly contain mesopores. However, the extraction conditions affect the porous structure of the polymer matrix and its extraction in the boiling solvent leads to the appearance of larger mesopores and a small fraction of macropores. This is caused by the deeper extraction of a macromolecules template as a result of swelling of polymer chains and an increase in their mobility.

**Figure 10:**
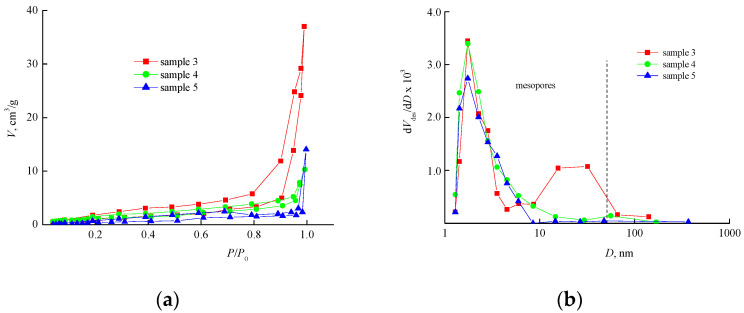
Adsorption-desorption isotherms (**a**) and pore size distribution curves (**b**) of the VP-PEGMMA-TEGDM polymer matrices after extraction of the polymer additive for 7 h (samples 3 and 5) and 3.5 h (sample 4).

Figure 10 shows the isotherms of nitrogen adsorption-desorption caused by the VP-PEGMMA-TEGDM polymer matrices; the hysteresis indicated the presence of mesopores in the studied samples. The pore size distribution curves show a pronounced peak of mesopores with a diameter of up to 10 nm. With an increase in the extraction time to 7 h, larger mesopores appear in sample 3, and the distribution becomes bimodal. The pore size distribution in these polymer matrices may be due to the high thermodynamic compatibility of the monomer mixture and the polymer additive, as well as to a decrease in the scale of the phase separation during the formation of the VP-PEGMMA-TEGDM polymer composite. This assumption is based on the DLS data related to light scattering of the corresponding monomer-copolymer mixtures (see Section 3.1).

According to the BET data, the VP-PEGMMA-TEGDM polymer matrices are characterized by significantly lower values of S_sp_ and V_p_ (Table 3). However, this may be due to the limits of this method in determining the surface characteristics of polar objects. Thus, the isotherm of the polymer matrix with the highest PEGMMA content (Figure 10a, sample 5) belongs to type III, according to the IUPAC classification, and is characteristic for the samples in which the adsorbate—adsorbate interaction is stronger than the adsorbate—adsorbent interaction [36]. This allows us to make propose the existence of a weak interaction of the nitrogen molecules with the polar matrix of the given composition.

In this regard, we tried to estimate the S_sp_ value of the polymer matrices based on the data of the Rose Bengal adsorption from aqueous solutions. For the relevant calculations, we used the values of the optical density of the RB solutions upon reaching equilibrium in the process of the RB adsorption by polymer matrices, as well as the value of the molar extinction coefficient of the aqueous RB solution. Specific experiments have revealed, that in the presence of br-VP-TEGDM, the molar extinction coefficient changes insignificantly.

According to the calculations, the S_sp_ value of the VP-TEGDM polymer matrix is around 21.4 m^2^/g (according to the BET data, S_sp_ = 22 m^2^/g), and the VP-PEGMMA-TEGDM polymer matrices (samples 3, 4) have S_sp_ values equal to 3.8 and 4.9 m^2^/g. The values obtained for these matrices are in good agreement with the BET analysis data (Table 3). An exception to this is the VP-PEGMMA-TEGDM polymer matrix with a high content of PEGMMA units (sample 5), for which S_sp_ = 8.3 m^2^/g, i.e., 4 times higher. Thus, according to the data obtained using these two methods, the VP-PEGMMA-TEGDM polymer matrices have a less developed specific surface area compared to the VP-TEGDM matrix. This can be associated with a change in the composition of the monomer-polymer mixtures and the copolymer additive characterized by high solubility and thermodynamic affinity in the corresponding mixtures.

### 3.6. Adsorption of Rose Bengal from Aqueous Solutions by Polymer Matrices

An important characteristic of MIPs is their affinity for a recognizable object, the ability to sorb it from a solution and release it during the extraction of MIPs with a solvent. Using RB as a model compound, we investigated the sorption properties of the obtained polymer matrices.

In the visible range of wavelengths, the absorption band of the dye in water is complex and reveals a superposition of two absorption bands at λ_max_ ~514 and 544 nm, which, respectively, belong to dimeric and monomeric forms of RB [37]. Depending on the pH, an equilibrium of several tautomeric forms of RB in aqueous solutions exists [38]. It is known [39] that the protonation of the phenolic group (xanthene ring) affects the chromophore part of the dye, providing more pronounced spectral changes in the UV-visible region than the protonation of the carboxylate group (benzene ring). The value of pK_a_ = 3.93 is assigned to the phenolic OH group in the xanthene ring, and pK_a_ = 1.89 is assigned to the carboxyl group [38]. In water solutions with pH values of 5–6, both RB groups are found to be deprotonated and are represented by RH^-^ and R^2-^ tautomeric forms, respectively, which is in equilibrium with the protonated forms. The charged particles can form complexes with substrate molecules, such as albumin [40]. The most effective interaction of RB with albumin occurs at pH < 5.0, when molecules of the protein are positively charged, and RB occurs in mono- or dianionic forms. However, the VP copolymers studied in this work do not contain ionic groups and, therefore, the formation of complexes via the ionic mechanism between copolymers and deprotonated form of dye is improbable.

**Figure 11:**
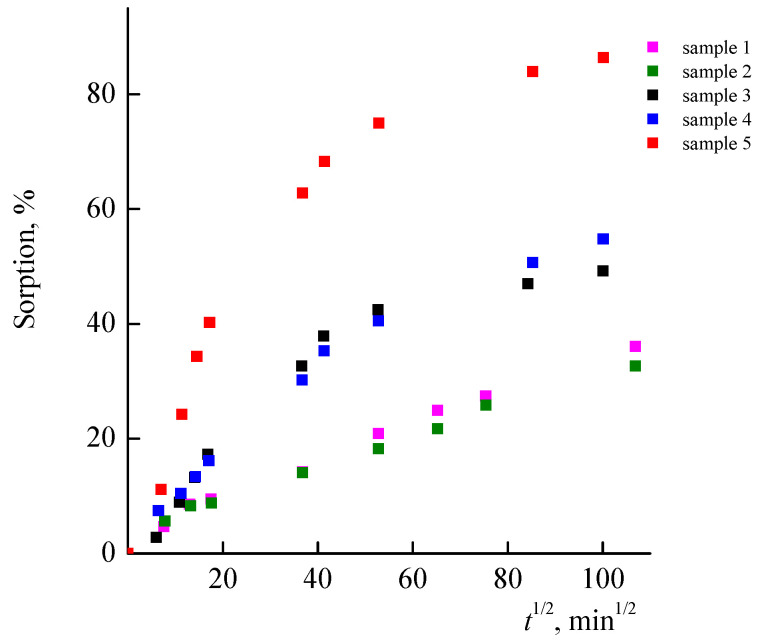
Dependences of RB adsorption from aqueous solutions by VP-TEGDM (samples 1, 2) and VP-PEGMMA-TEGDM (samples 3–5) polymer matrices.

Figure 11 shows the curves of RB adsorption by the VP-PEGMMA-TEGDM polymer matrices (samples 3–5), in the coordinates of the Fick equation. It can be seen that samples 3 and 4 exhibit similar properties and adsorb around 45–50% of the RB during 10,000 min (~7 days). The polymer matrix with a high content of PEGMMA units (sample 5) possesses a higher sorption capacity as the S_ads_ value for the RB adsorption from the aqueous solution is over 80%, which is a result of a high affinity of the dye to the polar matrix, as well as an increase in the number of adsorption centers such as the oxygen atoms in the ethylene oxide groups of PEGMMA units.

This assumption is supported by the curves of the RB adsorption from the aqueous solution by the VP-TEGDM polymer matrices with S_sp_ = 11 and 22 m^2^/g, which are significantly lower (Figure 11) compared with similar dependences for the VP-PEGMMA-TEGDM polymer matrices, i.e., the RB adsorption occurs at a slower rate. Moreover, the adsorption value does not depend on the specific surface area of matrices, and both samples have similar adsorption properties. Figure 8b shows a micrograph of the surface of the VP-TEGDM polymer matrix after RB adsorption and drying from water. It can be seen that its surface is developed, and the structure of the polymer matrix does not change.

The limiting values of adsorption for samples 3–5 are around 66, 74 and 91%, respectively, at 80 × 10^3^ min (56 days). In this case, the wavelength of the absorption band of RB in water shifts from 544 to 554, 552, and 557 nm, respectively. This may be due to the presence of stable associates (An^−^)_n_ in water. They are formed when dispersion interactions between the π-systems of the dye molecules, and the hydrophobic interactions, exceed electrostatic repulsion of like-charged particles [41].

### 3.7. Desorption of RB from Nanoporous Polymer Matrices

**Figure 12:**
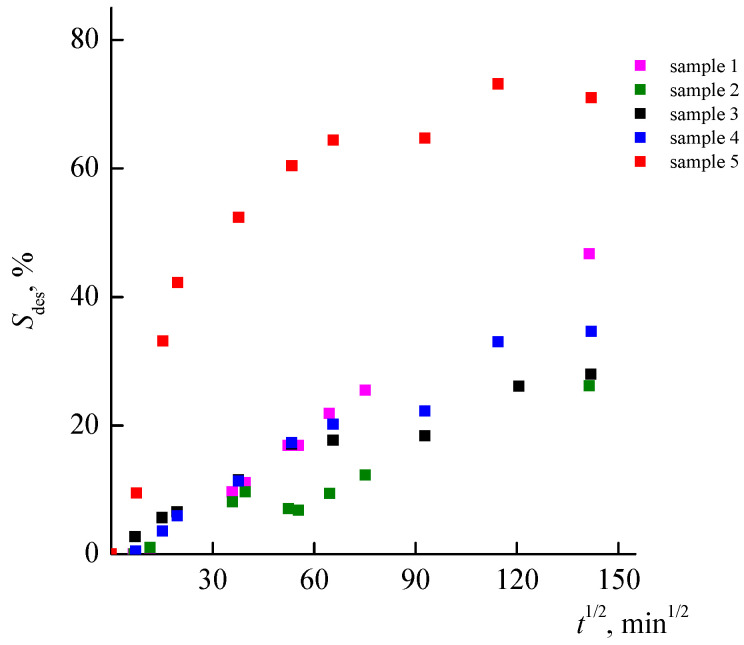
Dependences of RB desorption from the VP-TEGDM and VP-PEGMMA-TEGDM nanoporous polymer matrices of various compositions on time.

Figure 12 shows the curves of RB desorption from the VP-TEGDM and VP-PEGMMA-TEGDM polymer matrices. The RB desorption from samples 1–4 proceeds at close rates and is slow in nature. At first, the dye, which appears to be weakly bound to the polymer matrix, is washed out rather quickly after which the rate of the process slows down. The RB desorption from sample 5 proceeds more intensively: the relative RB amount released into the solution is around 70%, i.e., at almost twice the rate of samples 3 and 4. This may be due to stronger intermolecular interactions that exist between RB and these polymer matrices.

### 3.8. Electrochemical Behavior of Free and Bound RB in Aqueous Solution

**Figure 13:**
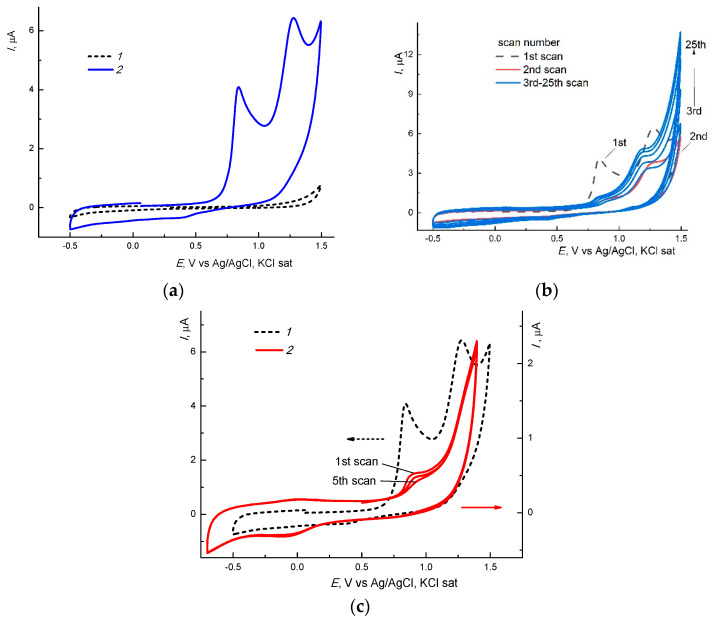
CVA-curves of oxidation of RB (~10^−3^ M) (**a**,**b**) and copolymer-bound RB (**c**) on a glassy carbon (GC) electrode (1 mm diameter) in PBS during the first (**a**) and subsequent (**b**) scans; *2*—2nd scan; *3*—3rd–10th scans. Dashed lines 1: (**a**)—background curve; (**b**,**c**)—curve *2* of Figure (**a**). v = 0.1 V ∙ s^−1^.

Due to its unique properties, RB is widely used [42], in photodynamic therapy [43], photo-supercapacitors [44] and for sensors [45], and it can be used to study a variety of methods such as photochemical [46] and electrochemical methods [46,47,48,49], etc. The electrochemical behavior of RB is rather complex, as it is oxidized in the potential E range of around +(0.5–1.5) V, demonstrating several irreversible waves of a different height on solid electrodes (gold, glassy carbon [46,47]). According to [47], the 1st RB oxidation wave is associated with the formation of a charge transfer complex, and the 2nd wave may be explained through its destruction. Electropolymerization could also occur under more positive potentials. Figure 13a, contains the 1st scan of the CVA-curve of RB on a GC electrode in a phosphate buffer solution (PBS), with pH 7.4. We observed a similar picture for Pt and Au. It is interesting to note that when the two waves of approximately equal height are observed on the 1st scan at an E of around +0.85 and +1.35, respectively, the CVA-curves on the 2nd and all subsequent scans are of a completely different form (cf. curves *1* and *2*–*3*, Figure 13b) as wave 1 sharply decreased in height and was barely visible, and the maximum observed of wave 2 disappears. Moreover, starting from the 3rd scan, the height of these waves progressively increases with the scan number. Similar curves were observed, in particular, during the oxidative polymerization of porphyrins [50] which indicate the formation of a conducting polymer film on an electrode.

A considerably different electrochemical behavior was exhibited by RB encapsulated into the branched VP-TEGDM copolymer, as shown in Figure 13c. It can be seen that the nature of the curves changes significantly: the first peak shifts to the region of more positive potentials, and the second one disappears entirely. These observations can be considered as evidences of the formation a copolymer-bound RB. The formation of such a structure alters the energetics of a copolymer-RB complex with respect to the initial RB. In part, the occurrence of the electrooxidation of the complex appears to be somewhat more difficult than that of RB. Similar behavior has been observed for the electroreduction of inclusive complexes of RB with cyclodextrines on mercury [48,49] and GC [43] electrodes whereby their reduction potentials shift, resulting in a lower E [48,49] and reduced heights, i.e., the I of the peaks decreases [43] with respect to RB. We have previously observed the considerable differences in electrochemical behavior between initial forms of depolarizers and those encapsulated into such copolymers, e.g., for the cases of zinc tetraphenylporphyrinate [51,52] and various complexes of Pt(IV) [53,54].

### 3.9. Quantum Chemical Modeling the Structure of H-Complex of RB with VP Monomer and VP-VP-VP Site of the Copolymer

**Figure 14:**
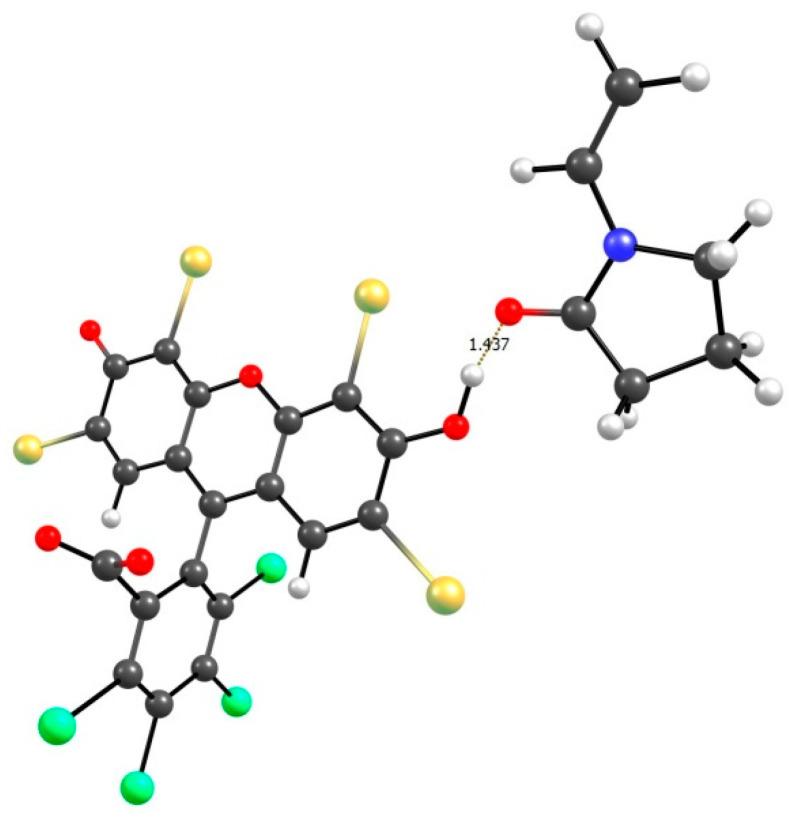
Structure of the most probable H-complex of VP monomer-RB.

Thus, the experimental data indicate the interaction of RB molecules with a VP copolymer. Quantum chemical calculations of the possible intermolecular complexes were carried out, with the VP monomer and the protonated form of RB used as an example. It has been shown that the most preferable structure is a bvpb1 structure (Figure 14), which is formed due to the bonding of the oxygen atom of the carbonyl group of VP with an OH group of the xanthene ring of the dye.

Other possible structures are provided in the Supplementary material (Figure S2), and the parameters of their H-bonds are provided in Table 4. From these data, it follows that the existence of structures bvpa1 (C=O(RB)... C-H_2_(VP)), bvpa2 ((C=O(RB)... C-H (VP)), bvpb2 (C=O (VP)... C-H (RB)), and bvpb3 (C=O (VP)... C-H (RB)) is extremely unlikely. In addition to the fact that the energies of intermolecular bonds (E_b_) are relatively small (from 1.63 to 2.82 kcal/mol), the enthalpies of formation of H-bonds (ΔH_f_) in these complexes have small absolute values (from 0.072 to −0.683 kcal/mol), although some of them are positive, which makes the reactions of the complex formation endothermic.

The enthalpy of formation of the bvpb1 complex is −9.07 kcal/mol, which making it possible to compare it with stable complexes such as the dimer of acetic acid (ΔH_f_ = −7.5 kcal/mol) or a complex of phenol with triethylamine (ΔH_f_ = −9 kcal/mol) [55]. As is known [56], the values of the electron density (ρ) of hydrogen bonds are in the range of 0.002–0.035 a.u. The obtained value ρ is 0.086677 a.u. This indicates, at least within the framework of the QTAIM theory, that the hydrogen bond is strong, with the contribution of covalent interactions.

An intermolecular interaction between the RB and the VP monomer is also evidenced by the absorption spectra of their aqueous solutions (Figure S3). It can be seen that, in the presence of the monomer, the maximum value of the absorption band of RB shifts by ~7 nm towards longer waves.

**Figure 15:**
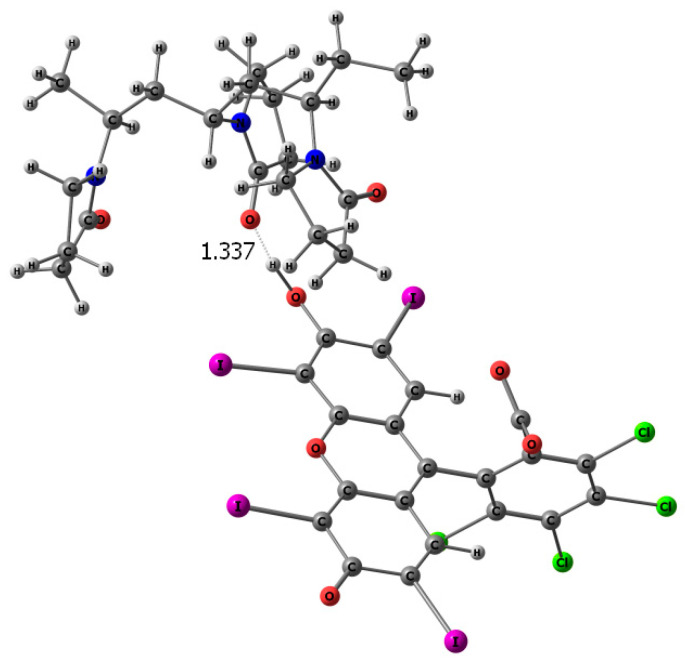
Optimized geometry (tpssh/lanl2dz) of a VP-VP-VP site of the copolymer with RB molecule.

Thus, a prepolymerization complex may be formed between the monomer and the hydroxyl group of RB; during polymerization, it may be fixed in certain positions of the rigid polymer matrix. Its extraction, using an appropriate solvent will lead to the appearance of molecular imprints in the polymer—with cavities complementary to the template in their size, shape, and in the arrangement of functional groups; the removal of the macromolecular porogen will lead to the appearance of pores in the matrix that provide access to molecular recognition sites. The re-binding of RB with the polymer in the course of molecular recognition will likely occur due to the same interactions, i.e., the hydrogen bond formation. The possibility of its formation is evidenced by the results of calculations of the complex between the hydroxyl-protonated form of RB and a site of three VP-VP-VP units of the copolymer (Figure 15). As in the prepolymerization complex, the bond between the oxygen atom of the C=O group of the lactam ring of the VP unit and the OH group of the dye is rather short and is even slightly stronger than in the complex of RB with the VP monomer. Thus, the formation of strong VPVPVP-RB complexes in the course of the molecular recognition of the dye by the MIP based on the developed network matrices is possible.

Amphiphilic polymer matrices, based on *N*-vinylpyrrolidone with a nanoporous structure, were prepared using three-dimensional radical polymerization in bulk in the presence of nanostructured objects as macromolecular porogens. During curing, the inert polymer additives separate into discrete regions as a result of the phase separation of the forming copolymer and the reaction mixture. After their extraction, using a thermodynamically suitable solvent, the stable pores of the nanometer range appear in the polymer matrix. The high degree of crosslinking of the polymer network provides their rigidity and mechanical stability. The resulting polymer matrices are capable of adsorbing hydrophilic dye molecules as the presence of PEGMMA units in the matrix and an increase in its content lead to the increase in the rate of RB adsorption. In all of the cases, desorption is found to be slow, which is associated with a slow destruction of the H-complexes formed between protonated RB molecules and oxygen-containing groups of the copolymers. The electron absorption spectroscopy and electrochemistry data also indicate an interaction between RB and the copolymers. Quantum chemical modeling has revealed that the most stable structures are H-complexes formed through the binding of the oxygen atom of the carbonyl group of VP and the OH-group of the xanthene ring of RB. The results of this study indicate that environmentally friendly and stable polymer matrices, based on *N*-vinylpyrrolidone, present potential as “smart” polymers for the recognition of hydrophilic dyes, such as fluorescein and its halogen derivatives—Rose Bengal, and potentially eosin and erythrosine, and from their removal from water as pollutants.

## Figures and Tables

**Table 1:** The molar composition, average molecular weight, polydispersity, glass transition temperature, and hydrodynamic radius of branched copolymers.

Copolymer	Composition of Copolymer, mol.%	*M*_w_ × 10^−3^	*p*	*T*_g_, °C	*R*_h_ *, nm
br-VP-TEGDM *	80.5:12.4:7.1	20.0	1.8	63.0	4.0
br-VP-PEGMMA- TEGDM	86.5:9.9:3.6	24.0 **	-	69.9	3.0

* the physicochemical data are presented in [24]; in 1% copolymer solution in isopropyl alcohol at 25 °C; ** determined from the light scattering data by the Debye method.

**Table 2:** Compositions and designations of monomer-polymer mixtures, polymer composites, and corresponding polymer matrices extracted by isopropyl alcohol at various conditions *.

Sample, NN	Monomer Mixture	Composition of Mixture, wt.%	Polymer Additive, 20 wt.%	Polymer Composite	Polymer Matrix
1	VP-TEGDM	40:0:60	br-VP-TEGDM	VP-TEGDM	VP-TEGDM
2					
3	VP-PEGMMA-TEGDM	40:10:50	br-VP-PEGMMA-TEGDM	VP-PEGMMA-TEGDM	VP-PEGMMA-TEGDM
4					
5		40:20:40			

* 1–7 h, 20 °C; 2–14 h, 80 °C; 3–7 h, 80 °C; 4–3.5 h, 80 °C; 5–7 h, 80 °C.

**Table 3:** Parameters of porous structure of the polymer networks according to the BET data.

Polymer Matrices	Composition of Monomer-Polymer Mixtures, wt%	Extraction Time, h	*S*_sp_, m^2^/g	*V*_p_, cm^3^/g
VP-TEGDM *	(40:60):20	7.0	11.0	0.026
		14.0	22.0	0.078
VP-PEGMMA- TEGDM **	(40:10:50):20	7.0	5.0	0.057
		3.5	4.5	0.016
	(40:20:40):20	7.0	2.0	0.011

* samples 1, 2; ** samples 3–5.

**Table 4:** Parameters of H-bond in the VP monomer-RB complexes.

Complexes	ρ(r), a.u.	∇^2^ ρ(r), a.u.	*E*_b_, kcal/mol	Δ*H*_f_, kcal/mol	Δ*H*_f_, kcal/mol (Excluding Energy of the Zero Vibrations)
bvpa1	+0.009799	+0.048321	−2.12	0.072	−1253
bvpa2	+0.008217	+0.039760	−1.63	0.201	−1.105
bvpb1	+0.086677	+0.175948	−31.13	−9.07	−9.625
bvpb2	+0.011927	+0.060106	−2.82	−0.604	−1.835
bvpb3	+0.011781	+0.052662	−2.57	−0.683	−1.987

## Data Availability

The data presented in this study are available on request from the corresponding author.

## Supplementary Materials

The following are available online at https://www.mdpi.com/article/10.3390/ma14226757/s1: Description of determination diffusion and sorption properties; Figure S1: Dependence of sorption of water vapor S by VP-TEGDM polymer composite and corresponding polymer matrix after extraction of copolymer additive on time; Figure S2: 3D-structures of the VP monomer-RB complex; Figure S3: Absorption spectra of aqueous RB solution in the absence and in the presence of the VP monomer. The RB concentration in water is 2.5 × 10^−5^ M; the RB concentration in the water-VP mixture is 2.4 × 10^−5^ M; Table S1: Content of sol and gel fractions in VP-PEGMMA-TEGDM polymer composites.

## References

[1] Wulff G., Sarhan A. (1972). Use of polymers with enzyme-analogous structures for the resolution of racemates. Angew. Chem..

[2] Arshady R., Mosbach M. (1981). Synthesis of substrate-selective polymers by host-guest polymerization. Macromol. Chem. Chem. Phys.-Macromol. Chem..

[3] Hasanah A.N., Soni D., Pratiwi R., Rahayu D., Megantara S., Mutakin (2020). Synthesis of diazepam-imprinted polymers with two functional monomers in chloroform using a bulk polymerization method. J. Chem..

[4] Keçili R., Hussain C.M. (2018). Recent progress of imprinted nanomaterials in analytical chemistry. Int. J. Anal. Chem..

[5] Dinc M., Esen C., Mizaikoff B. (2019). Recent advances on core–shell magnetic molecularly imprinted polymers for biomacromolecules. TrAC-Trends Anal. Chem..

[6] Piletsky S., Canfarotta F., Poma A., Bossi A.M., Piletsky S. (2020). Molecularly imprinted polymers for cell recognition. Trends Biotechnol..

[7] Muratsugu S., Shirai S., Tada M. (2020). Recent progress in molecularly imprinted approach for catalysis. Tetrahedron Lett..

[8] Mokhtari P., Ghaedi M. (2019). Water compatible molecularly imprinted polymer for controlled release of riboflavin as drug delivery system. Eur. Polym. J..

[9] Lulinski P. (2017). Molecularly imprinted polymers based drug delivery devices: A way to application in modern pharmacotherapy. A review. Mater. Sci. Eng. C.

[10] Yang Y.K., Yan W.Y., Guo C.X., Zhang J.H., Yu L.G., Zhang G.H., Wang X.M., Fang G.Z., Sun D.D. (2020). Magnetic molecularly imprinted electrochemical sensors: A review. Anal. Chim. Acta.

[11] Huang D.L., Tang Z.H., Peng Z.W., Lai C., Zeng G.M., Zhang C., Xu P.A., Cheng M., Wan J., Wang R.Z. (2017). Fabrication of water-compatible molecularly imprinted polymer based on β-cyclodextrin modified magnetic chitosan and its application for selective removal of bisphenol A from aqueous solution. J. Taiwan Inst. Chem. Eng..

[12] Zhang Y., Li Y.W., Hu Y.L., Li G.K., Chen Y.Q. (2010). Preparation of magnetic indole-3-acetic acid imprinted polymer beads with 4-vinylpyridine and beta-cyclodextrin as binary monomer via microwave heating-initiated polymerization and their application to trace analysis of auxins in plant tissues. J. Chromatogr. A.

[13] Urraca J.L., Hall A.J., Moreno-Bondi M.C., Sellergren B. (2006). A Stoichiometric Molecularly imprinted polymer for the class-selective recognition of antibiotics in aqueous media. Angew. Chem. Int. Ed..

[14] Oral E., Peppas N.A. (2006). Hydrophilic Molecularly Imprinted poly(hydroxyethyl-methacrylate) polymers. J. Biomed. Mater. Res. A.

[15] Manesiotis P., Borrelli C., Aureliano C.S.A., Svensson C., Sellergren B. (2009). Water-Compatible Imprinted Polymers for Selective Depletion of Riboflavine from Beverages. J. Mater. Chem..

[16] Kubo T., Hosoya K., Nomachi M., Tanaka N., Kaya K. (2005). Preparation of a Novel Molecularly Imprinted Polymer Using a Water-Soluble Crosslinking Agent. Anal. Bioanal. Chem..

[17] Sarpong K.A., Xu W., Huang W., Yang W. (2019). The development of molecularly imprinted polymers in the clean-up of water pollutants: A review. Amer. J. Analyt. Chem..

[18] Wu D., Xu F., Sun B., Fu R., He H., Matyjaszewski K. (2012). Design and preparation of porous polymers. Chem. Rev..

[19] Nemanash M., Noh J.-H., Meijboom R. (2018). Dendrimers as alternative templates and pore-directing agents for the synthesis of micro- and mesoporous materials. J. Mater. Sci..

[20] Kurmaz S.V., Kochneva I.S., Perepelitsina E.O., Bubnova M.L., Bakova G.M., Knerel’man E.I., Davydova G.I. (2013). Ethyl acrylate copolymers as promising porogens for the synthesis of polydimethacrylates with controlled porous structures. Polym. Sci..

[21] Kurmaz S.V., Grubenko G.A., Knerelman E.I., Davydova G.I., Torbov V.I., Dremova N.N. (2014). Promising macromolecular nanoobjects for the template synthesis of network copolymers with mesoporous structure. Mendeleev Commun..

[22] Fadeeva N.V., Kurmaz S.V., Knerelman E.I., Davydova G.I., Torbov V.I., Dremova N.N. (2016). New polymer materials with controlled nanoporous structure based on N-vinylpyrrolidone. Russ. Chem. Bull..

[23] Fadeeva N.V., Kurmaz S.V., Knerelman E.I., Davydova G.I., Torbov V.I., Dremova N.N. (2017). Nanoporous polymer networks based on N-vinylpyrrolidone. Polym. Sci. Ser. B.

[24] Kurmaz S.V., Fadeeva N.V., Knerelman E.I., Davydova G.I., Torbov V.I., Dremova N.N. (2019). Nanoporous polymer networks of *N*−vinylpyrrolidone with dimethacrylates of various polarity. Synthesis, structure, and properties. J. Polym. Res..

[25] Kurmaz S.V., Pyryaev A.N. (2010). Synthesis of *N*-vinyl-2-pyrrolidone-based branched copolymers via crosslinking free-radical copolymerization in the presence of a chain-transfer agent. Polym. Sci..

[26] Frisch M.J., Trucks G.W., Schlegel H.B., Scuseria G.E., Robb M.A., Cheeseman J.R., Scalmani G., Barone V., Mennucci B., Petersson G.A. (2009). Gaussian 09, Revision, B.01.

[27] Tao J.M., Perdew J.P., Staroverov V.N., Scuseria G.E. (2003). Climbing the density functional ladder: Nonempirical meta–generalized gradient approximation designed for molecules and solids. Phys. Rev. Lett..

[28] Kurmaz S.V., Fadeeva N.V., Ignat’ev V.M., Kurmaz V.A., Kurochkin S.A., Emel’yanova N.S. (2020). Structure and state of water in branched *N*-vinylpyrrolidone copolymers as carriers of a hydrophilic biologically active compound. Molecules.

[29] Todd A., Keith T.K. (2010). AIMAll, Version 10.05.04.

[30] Espinosa E., Molins E., Lecomte C. (1998). Hydrogen bond strengths revealed by topological analyses of experimentally observed electron densities. Chem. Phys. Lett..

[31] Chemcraft—Graphical Software for Visualization of Quantum Chemistry Computations. https://www.chemcraftprog.com.

[32] Kurmaz S.V., Fadeeva N.V., Knerelman E.I., Davydova G.I. (2018). Preparation of porous polymer networks of *N*-vinylpyrrolidone with triethylene glycol dimethacrylate and determination of their specific surface area using Rose Bengal dye. Russ. J. Appl. Chem..

[33] Vlasova I.M., Polyansky D.V., Vlasov A.A., Saletsky A.M. (2013). Investigation of rotational diffusion of the Rose Bengal fluorescent nanomarker in human serum albumin solutions. Mosc. Univ. Phys. Bull..

[34] Roshchupkin V.P., Kurmaz S.V. (2004). State-of-the-art in the studies of three-dimensional radical copolymerization. Uspekhi Khimii.

[35] Vlakh E.G., Korzhikov V.A., Hubina A.V., Tennikova T.B. (2015). Moleclar imprinting: A tool of modern chemistry for the preparation of highly selective monolithic sorbents. Russ. Chem. Rev..

[36] Gregg S.J., Sing K.S.W. (1982). Adsorption, Surface Area and Porosity.

[37] Fini P., Catucci M., Castagnolo M., Cosma P., Pluchinotta V., Agostiano A. (2007). Spectroscopic investigation of Rose Bengal/cyclodextrin interactions in aqueous solution: The case of the hydroxypropyl-cyclodextrins. J. Phenom. Macrocycl. Chem..

[38] Batistela V.R., Pellosi D.S., de Souza F.D., da Costa W.F., de Oliveira Santin S.M., de Souza V.R., Caetano W., Moisés de Oliveir H.P., Scarminio I.S., Hioka N. (2011). pKa determinations of xanthene derivates in aqueous solutions by multivariate analysis applied to UV–Vis spectrophotometric data. Spectrochim. Acta.

[39] Ueno T., Urano Y., Setsukinai K., Takakusa H., Kojima H., Kikuchi K., Ohkubo K., Fukuzumi S., Nagano T. (2004). Rational principles for modulating fluorescence properties of fluorescein. J. Am. Chem. Soc..

[40] Vlasova I.M., Vlasova A.A., Kuleshova A.A., Gordeyeva Y.A., Saletskiy A.M. (2020). Constants of the Formation of Complexes with Nanomarkers of the Fluorescein Family and Bovine Serum Albumin in Aqueous Solutions. Russ. J. Phys. Chem. A.

[41] Ishchenko A.A., Shapovalov S.A. (2004). Heterogeneous Association of the Ions of Dyes in Solutions. J. Appl. Spectrosc..

[42] Demartis S., Obinu A., Gavini E., Giunchedi P., Rassu G. (2021). Nanotechnology-based rose Bengal: A broad-spectrum biomedical tool. Dye. Pigment..

[43] Alexandrino F.J.R., Bezerra E.M., Da Costa R.F., Cavalcante L.R.L., Sales F.A.M., Francisco T.S., Rodrigues L.K.A., de Brito D.H.A., Ricardo N.M.P.S., Costa S.N. (2019). Rose Bengal incorporated to alpha-cyclodextrin microparticles for photodynamic therapy against the cariogenic microorganism Streptococcus mutans. Photodiagnosis Photodyn. Ther..

[44] Das A., Deshagani S., Ghosal P., Deepa M. (2020). Redox active and electrically conducting cobalt telluride Nanorods/Poly(1-aminoanthraquinone) composite and photoactive Rose Bengal dye based photo-supercapacitor. Appl. Mater. Today.

[45] Midya A., Ghosh R., Santra S., Ray S.K., Guha P.K. (2016). Reduced graphene oxide-rose bengal hybrid film for improved ammonia detection with low humidity interference at room temperature. Mater. Res. Express.

[46] Linden S.M., Neckers D.C. (1988). Bleaching studies of rose-bengal onium salts. J. Am. Chem. Soc..

[47] Andrieux F.P.L., Boxall C. (2007). Electrochemical studies of Rose Bengal using the electrochemical quartz crystal microbalance. ECS Trans..

[48] Fini P., Longobardi F., Catucci L., Cosma P., Agostiano A. (2004). Spectroscopic and electrochemical study of Rose Bengal in aqueous solutions of cyclodextrins. Bioelectrochemistry.

[49] Fini P., Loseto R., Catucci L., Cosma P., Agostiano A. (2007). Study on the aggregation and electrochemical properties of Rose Bengal in aqueous solution of cyclodextrins. Bielectrochemistry.

[50] Konev D.V., Lizgina K.V., Istakova O.I., Baulin V.E., Kalashnikova I.P., Devillers C.H., Vorotyntsev M.A. (2016). Electropolymerization of magnesium 5, 15-di (N-methoxyphenyl) porphine. Russ. J. Electrochem..

[51] Kurmaz S.V., Gak V.Y., Kurmaz V.A., Konev D.V. (2018). Preparation and Properties of Hybrid Nanostructures of Zinc Tetraphenylporphyrinate and an Amphiphilic Copolymer of N-Vinylpyrrolidone in a Neutral Aqueous Buffer Solution. Russ. J. Phys. Chem. A.

[52] Kurmaz S.V., Konev D.V., Sen’ V.D., Kurmaz V.A., Kulikov A.V. Preparation and characterization of stable water soluble hybrid nanostructures of hydrophobic compounds by encapsulation into nanoparticles of amphiphilic N-vinylpyrrolidone copolymers of new generation. Proceedings of the IOP Conference Series: Materials Science and Engineering.

[53] Kurmaz S.V., Fadeeva N.V., Fedorov B.S., Kozub G.I., Emel’yanova N.S., Kurmaz V.A., Manzhos R.A., Balakina A.A., Terentyev A.A. (2020). New antitumor hybrid materials based on Pt^IV^ organic complex and polymer nanoparticles consisting of N-vinylpyrrolidone and (di)methacrylates. Mendeleev Commun..

[54] Kurmaz S.V., Fadeeva N.V., Fedorov B.S., Kozub G.I., Kurmaz V.A., Ignat’ev V.M., Emel’yanova N.S. (2021). Amphiphilic copolymers of N-vinylpyrrolidone with (di)methacrylates as promising carriers for the platinum(IV) complex with antitumor activity. Russ. Chem. Bull..

[55] Maréchal Y. (2007). The Hydrogen Bond and the Water Molecule.

[56] Koch U., Popelier P.L.A. (1995). Conformational dependence of atomic multipole moments. J. Phys. Chem..

