# Supplementary material for: Mesoporous Networks of N-Vinylpyrrolidone with (di)Methacrylates as Precursors of Ecological Molecular Imprinted Polymers
Source: Materials (Basel). 2021 Nov 9;14(22):6757. doi: 10.3390/ma14226757 (PMC8625661; doi:10.3390/ma14226757)
Supplement: Supplementary file 1 [file materials-14-06757-s001.zip › materials-1417902-supplementary.pdf]

materials

MDPI

Supplementary Material

Mesoporous Networks of *N*-Vinylpyrrolidone with (di)Methacrylates as Precursors of Ecological Molecular Imprinted Polymers

Svetlana V. Kurmaz [1,*], Natalia V. Fadeeva [1], Anna I. Gorshkova [1,2], Sergey A. Kurochkin [1,3], Eugenia I. Knerelman [1], Galina I. Davydova [1], Vladimir I. Torbov [1], Nadezhda N. Dremova [1], Dmitry V. Konev [1], Vladimir A. Kurmaz [1], Vladislav M. Ignatiev [1,2] and Nina S. Emelyanova [1]

[1] Institute of Problems of Chemical Physics, ac. Semenov av., 1, Moscow region, 142432 Chernogolovka, Russia; natali-vi@inbox.ru (N.V.F.); anhen.gor@mail.ru (A.I.G.); oligo@icp.ac.ru (S.A.K.); kge@icp.ac.ru (E.I.K.); roxen67@mail.ru (G.I.D.); torbov@icp.ac.ru (V.I.T.); dremova@icp.ac.ru (N.N.D.); dkfrvzh@gmail.com (D.V.K.); kurmaz@icp.ac.ru (V.A.K.); ignvm@74.ru (V.M.I.); n_emel@mail.ru (N.S.E.)

[2] Institute of Problems of Chemical Physics, ac. Semenov av., 1, Moscow region, 142432 Chernogolovka, Russia, M.V. Lomonosov Moscow State University, Lenin Hills, 1, 119991 Moscow, Russia

[3] Institute of Problems of Chemical Physics, ac. Semenov av., 1, Moscow region, 142432 Chernogolovka, Russia, Bauman Moscow State Technical University, 2nd Baumanskaya st., 5, 105005 Moscow, Russia

* Correspondence: skurmaz@icp.ac.ru; Tel.: +7-496-522-10-89

Citation: Kurmaz, S.V.; Fadeeva, N.V.; Gorshkova, A.I.; Kurochkin, S.A.; Knerelman, E.I.; Davydova, G.I.; Torbov, V.I.; Dremova, N.N.; Konev, D.V.; Kurmaz, V.A.; et al. Mesoporous Networks of *N*-Vinylpyrrolidone with (di)Methacrylates as Precursors of Ecological Molecular Imprinted Polymers. *Materials* **2021**, *14*, 6757. https://doi.org/10.3390/ma14226757

Academic Editor: Marek Krzeminski

Received: 27 September 2021
Accepted: 4 November 2021
Published: 9 November 2021

Publisher's Note: MDPI stays neutral with regard to jurisdictional claims in published maps and institutional affiliations.

Copyright: © 2021 by the authors. Licensee MDPI, Basel, Switzerland. This article is an open access article distributed under the terms and conditions of the Creative Commons Attribution (CC BY) license (http://creativecommons.org/licenses/by/4.0/).

Description of determination diffusion and sorption properties; Figure S1. Dependence of sorption of water vapor S by VP-TEGDM polymer composite and corresponding polymer matrix after extraction of copolymer additive on time; Figure S2: 3D-structures of the VP monomer-RB complex; Figure S3: Absorption spectra of aqueous RB solution in the absence and in the presence of the VP monomer. The RB concentration in water is 2.5×10^{-5} M; the RB concentration in the water-VP mixture is 2.4×10^{-5} M; Table S1: Content of sol and gel fractions in VP-PEGMMA-TEGDM polymer composites

Diffusion and sorption properties. The diffusion and sorption properties of the VP-TEGDM polymer composite, and the porous matrix were investigated in water vapor at atmospheric pressure and room temperature (20 °C). Cylindrical samples of copolymers with a size of ~ 5 × 3 mm were used.

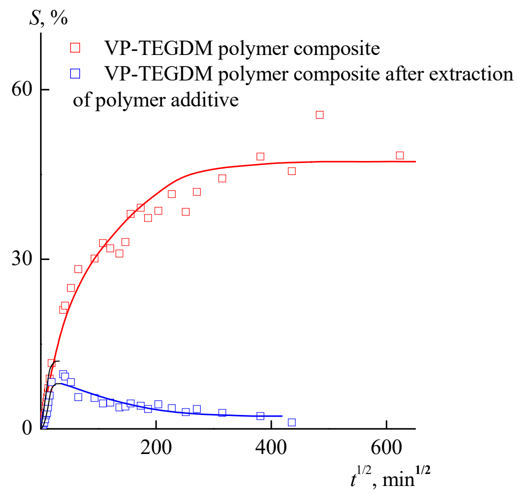

Figure S1. Dependence of sorption of water vapor S by VP-TEGDM polymer composite and corresponding polymer matrix after extraction of copolymer additive on time, 20 °C.

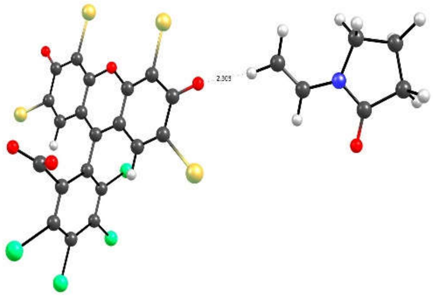

bvpa1

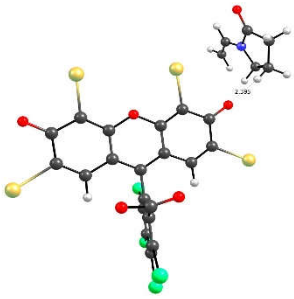

bvpa2

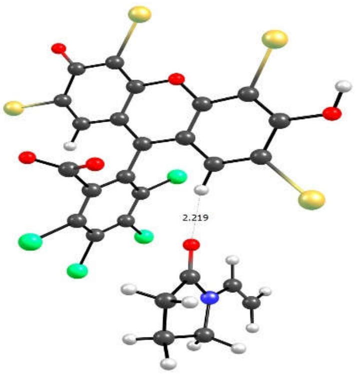

bvpb2

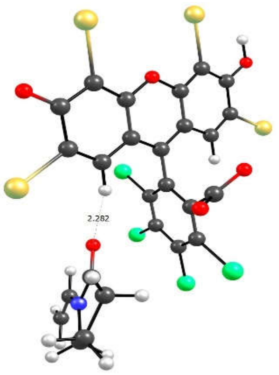

bvpb3

Figure S2. 3D-structures of the VP monomer-RB complex.

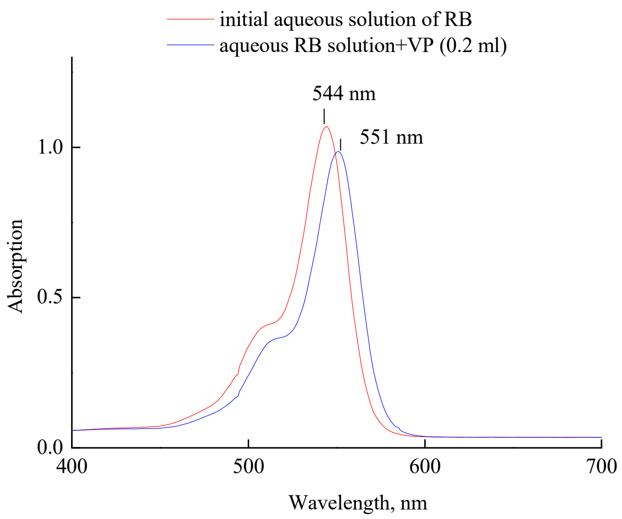

Figure S3. Absorption spectra of aqueous RB solution in the absence and in the presence of the VP monomer. The RB concentration in water is 2.47 × 10^{-5} M; the RB concentration in the water-VP mixture is 2.35 × 10^{-5} M.

Table S1. Content of sol and gel fractions in VP-PEGMMA-TEGDM polymer composites.

**Polymer composites**	**Composition of VP-PEGMMA -TEGDM – br-copolymer mixture, wt%**	**Extraction time, h**	**Gel content*, %**	**Sol content, %**	**The total content of gel and sol, %**
VP-PEGMMA -TEGDM	(40 : 10 : 50) : 20	7.0	86.0	18.0	104.0
		3.5	79.0	22.0	101.0
	(40 : 20 : 40) : 20	7.0	80.0	30.0	110.0
		3.5	85.0	26.0	111.0

* The theoretical content of the gel and the sol isolated from the polymer composites corresponds to 80 and 20 wt%.
